# High‐Frequency Ultrasound Assessment of Female Genital Rejuvenation

**DOI:** 10.1002/jum.70306

**Published:** 2026-08-09

**Authors:** Luciana Zattar, Natália Venturelli Marques de Souza

**Affiliations:** ^1^ Hospital Sírio‐Libanês São Paulo Brazil; ^2^ Clínica Venturelli São Paulo Brazil

**Keywords:** absorbable threads, aesthetic gynecology, calcium hydroxyapatite, female genital rejuvenation, high‐frequency ultrasound, hyaluronic acid

## Abstract

**Objectives:**

To describe the role of high‐frequency ultrasound (HFUS) in the anatomical evaluation and monitoring of female genital rejuvenation procedures and to illustrate characteristic sonographic patterns associated with commonly used aesthetic treatments.

**Methods:**

This retrospective observational study reviewed anonymized clinical and ultrasound records from eight female patients who underwent genital rejuvenation in a private dermatologic ultrasound center. HFUS examinations were performed using 20–33 MHz linear probes in standardized B‐mode, with color Doppler added when indicated. Representative cases were selected to demonstrate normal anatomy, age‐related changes, postprocedural findings, and complications.

**Results:**

HFUS provided high‐resolution visualization of the epidermis, dermis, subcutaneous tissue, and fascial planes of the external genitalia. Younger patients showed compact fat lobules and well‐defined fascial layers, whereas older patients exhibited dermal thinning and a subepidermal low‐echogenicity band. Postprocedural HFUS identified distinct patterns for each treatment modality, including anechoic lobulated deposits after hyaluronic acid, hyperechoic punctate “snowfall” reflections after calcium hydroxyapatite, linear hyperechoic structures after absorbable threads, and focal hyperechoic panniculitis following lipolytic mesotherapy. HFUS also aided in detecting and characterizing treatment‐related changes and potential complications.

**Conclusions:**

HFUS appears to be a useful adjunct for minimally invasive female genital rejuvenation, enabling detailed anatomical assessment, visualization of injected materials, and monitoring of treatment‐related changes. These preliminary findings support the integration of HFUS into aesthetic practice and highlight the need for further studies to standardize its application in this growing field.

**Abbreviations**
B‐mode: brightness mode
CaHA: calcium hydroxyapatite
HA: hyaluronic acid
HFUS: high‐frequency ultrasound
PDO: polydioxanone
PLLA: poly‐L‐lactic acid
PMMA: polymethyl methacrylate
SLEB: subepidermal low‐echogenic band
SMAS: superficial musculoaponeurotic system

The practice of conducting surgical procedures on female genitalia is not a contemporary phenomenon; however, it was not until the 1970s that such interventions were first proposed for aesthetic considerations.^1^ In recent years, there has been a notable increase in the prevalence of procedures designed to enhance the female intimate area, which includes not only surgical techniques but also minimally invasive aesthetic methods, all aimed at improving sexual, functional, and aesthetic dimensions.^2^

Women are exhibiting an increasing interest in the enhancement of their intimate regions, a trend that can be attributed to a variety of factors, including the amplified visibility of the region through media portrayals, sexting, and the exchange of nude imagery, as well as shifting societal norms concerning hair removal and the persisting sexual activity among women post‐menopause, among other influences.^1^, ^3^, ^4^ This growing interest reflects a broader societal acceptance of discussing and addressing female genital aesthetics, paralleling trends observed in cosmetic gynecology.^5^

Minimally invasive genital rejuvenation procedures, such as radiofrequency, laser, injectable fillers, and absorbable threads, have emerged as alternatives to traditional labiaplasty, offering shorter recovery times and fewer complications.^6^, ^7^

Among these modalities, absorbable polydioxanone (PDO) threads and injectable biostimulators such as calcium hydroxyapatite (CaHA) and hyaluronic acid (HA) have demonstrated the ability to restore volume, improve skin turgor, and induce neocollagenesis.^4^, ^8^, ^9^ Recent reports have also described innovative HA injection techniques for labia majora rejuvenation, showing favorable aesthetic outcomes and patient satisfaction.^10^

Despite their increasing clinical adoption, there is limited imaging evidence describing the normal anatomy, procedural characteristics, and post‐treatment tissue behavior in the female genital region.

In this context, high‐frequency ultrasound (HFUS) represents a valuable diagnostic and monitoring tool, offering high‐resolution visualization of the epidermis, dermis, subcutaneous layers, and fascial planes. HFUS can identify prior interventions, guide filler or thread placement, and detect complications or anatomical variations that may affect procedural planning.

Accordingly, the present study aims to describe the role of high‐frequency ultrasound (HFUS) in female genital rejuvenation by illustrating its application in preprocedural assessment, intraprocedural guidance, postprocedural evaluation, and complication management.

## Material and Methods

### Study Design

This was a retrospective, descriptive, observational study performed at a private dermatologic ultrasound reference center in São Paulo, Brazil, specialized in high‐frequency imaging for aesthetic and genital procedures.

### Population

Clinical and imaging records of female patients who underwent genital rejuvenation procedures between January 2020 and June 2024 were reviewed. Inclusion criteria required availability of complete clinical documentation and high‐quality ultrasound images. Patients treated with injectable fillers such as hyaluronic acid (HA), calcium hydroxyapatite (CaHA), and poly‐L‐lactic acid (PLLA), as well as absorbable polydioxanone (PDO) threads and lipolytic mesotherapy were included in the analysis. Cases of complications, such as nodules, granulomas, panniculitis, hidradenitis, and neoplastic lesions, were also analyzed. All data were anonymized prior to analysis.

### Imaging Protocol

All examinations were performed using high‐frequency linear probes ranging from 20 to 33 MHz (Aplio i800, Canon Medical Systems Corporation, Japan). Standardized B‐mode imaging was performed in all cases, with additional compression and color Doppler evaluation when indicated. Preprocedural assessment focused on layered vulvar anatomy and the identification of previous interventions. Intraprocedural and postprocedural scans documented filler distribution, thread positioning, tissue remodeling, and material integration over time.

All ultrasound examinations were performed and interpreted by a single radiologist specialized in dermatologic and aesthetic ultrasound.

### Data Analysis

Clinical and imaging data were retrospectively retrieved and correlated with procedural technique and clinical outcome. Representative cases were selected to illustrate typical findings of female genital rejuvenation under HFUS, including normal anatomy, procedural applications, and complications.

### Ethical Considerations

The study was conducted in accordance with the principles of the Declaration of Helsinki. All patients provided consent for the use of their anonymized clinical data and images for research and publication purposes.

## Results

A total of eight anonymized female patients were analyzed through high‐frequency ultrasound (HFUS) imaging. Representative cases were selected to illustrate normal genital anatomy, age‐related structural variations, and findings associated with aesthetic procedures and complications. The following narrative describes the main imaging patterns observed.

### Anatomy and Baseline Assessment

**Figure 1:**
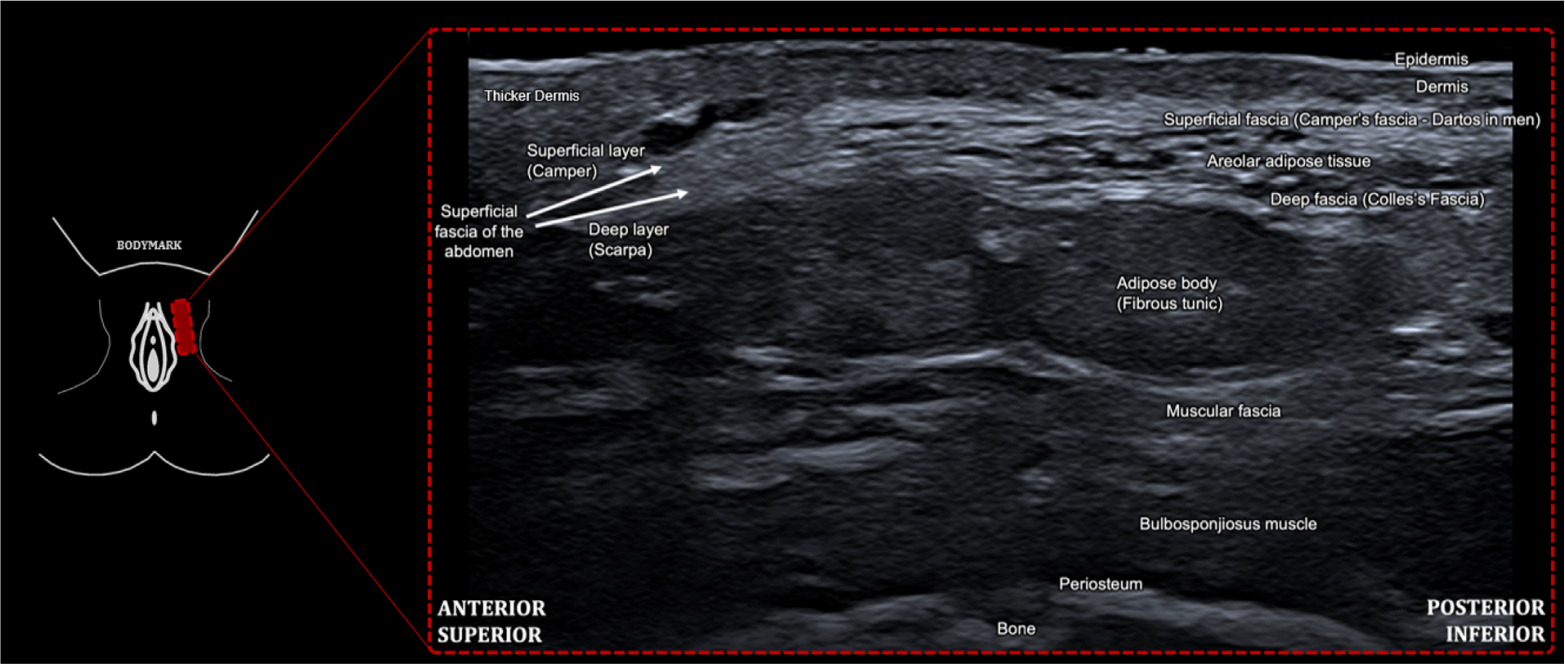
Normal anatomy of the female genital region. B‐mode 24 MHz image of a 34‐year‐old patient showing the well‐defined layered structure of the labia majora, including the epidermis, dermis, subcutaneous tissue, and fascial planes (Camper's and Scarpa's fascia).

HFUS enabled high‐resolution visualization of the external female genital structures, including the epidermis, dermis, subcutaneous tissue, and fascial planes. The epidermis appeared as a continuous hyperechoic line, while the dermis showed a uniform echogenic band corresponding to collagen fibers. Subcutaneous tissue was characterized by hypoechoic fat lobules separated by thin echogenic septa representing Camper's and Scarpa's fascia (Figure 1). Vascular mapping with color Doppler consistently demonstrated the anterior and posterior pudendal branches and their anastomoses.

The main anatomical regions evaluated and their sonographic features are summarized in Table 1, which also highlights the comparative differences between younger and aging patients.

**Table 1:** Summary of normal and age‐related ultrasound findings in the female genital region.

Region	HFUS appearance (younger)	HFUS appearance (aging)	Clinical relevance
Mons pubis	Compact fat lobules with thin echogenic septa; clear separation between Camper's and Scarpa's fascia.	Looser lobules and less defined fascial planes; dermal thinning and reduced echogenicity.	Reference for lipolytic procedures and volumetric restoration.
Labia majora	Thick dermis, organized collagen, homogeneous subcutaneous tissue; visible round ligament.	Decreased dermal reflectivity, irregular septa, and subcutaneous atrophy.	Main target for fillers and biostimulators.
Labia minora	Thin mucocutaneous fold with variable keratinization; rich vascular network on Doppler.	Slight dermal thinning; vascular flow less evident superficially.	Relevant for vascular safety during procedures.
Clitoral complex	Echogenic suspensory ligament; isoechoic erectile tissue of bulbs and crura.	No significant change in echogenicity; mild connective thinning.	Landmark for avoiding vascular or neural injury.
Perineal region	Echogenic fibromuscular layers; identifiable pudendal branches on Doppler.	Similar architecture, but with decreased superficial signal intensity.	Used for thread placement and vascular safety mapping.

Note: HFUS, high‐frequency ultrasound.

### Age‐Related Changes

**Figure 2:**
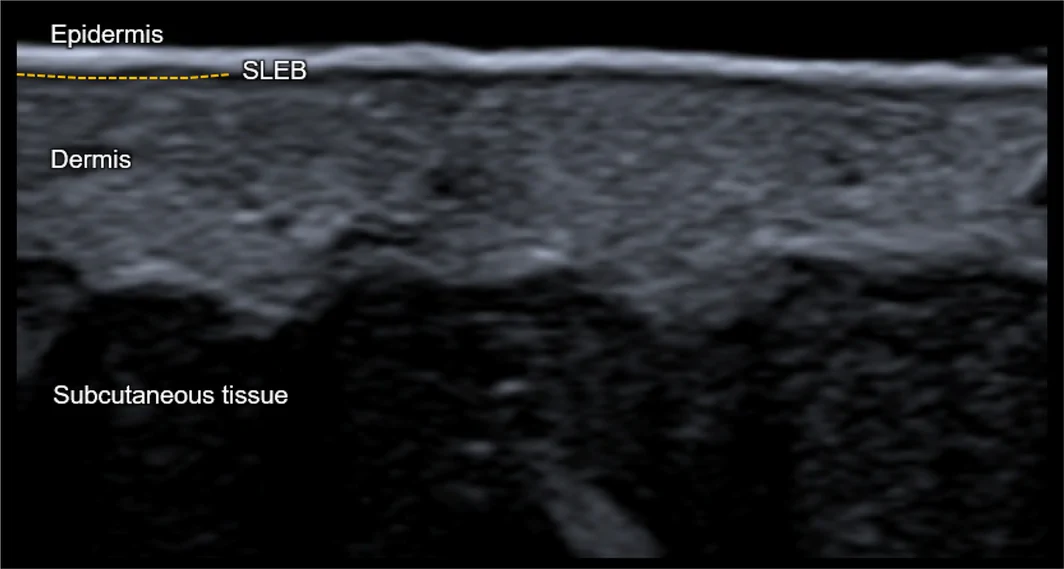
Age‐related changes in the female genital skin. B‐mode 33 MHz sagittal image of a 45‐year‐old patient demonstrating dermal thinning, decreased echogenicity, and the presence of a subepidermal low‐echogenicity band (SLEB, yellow dashed line).

HFUS revealed progressive structural modifications associated with chronological and hormonal aging. The dermis appeared thinner and less echogenic, with partial loss of collagen organization. A subepidermal low‐echogenicity band (SLEB) was consistently observed in aging patients, appearing as a hypoechoic layer immediately beneath the epidermis (Figure 2). The subcutaneous tissue also showed decreased compactness and reduced definition of fibrous septa, particularly in the mons pubis and labia majora.

### Procedural Findings

#### Hyaluronic Acid

Hyaluronic acid (HA) fillers are primarily indicated for labia majora hypotrophy, loss of volume, and reduced hydration, particularly in postmenopausal women or those with aesthetic concerns.

**Figure 3:**
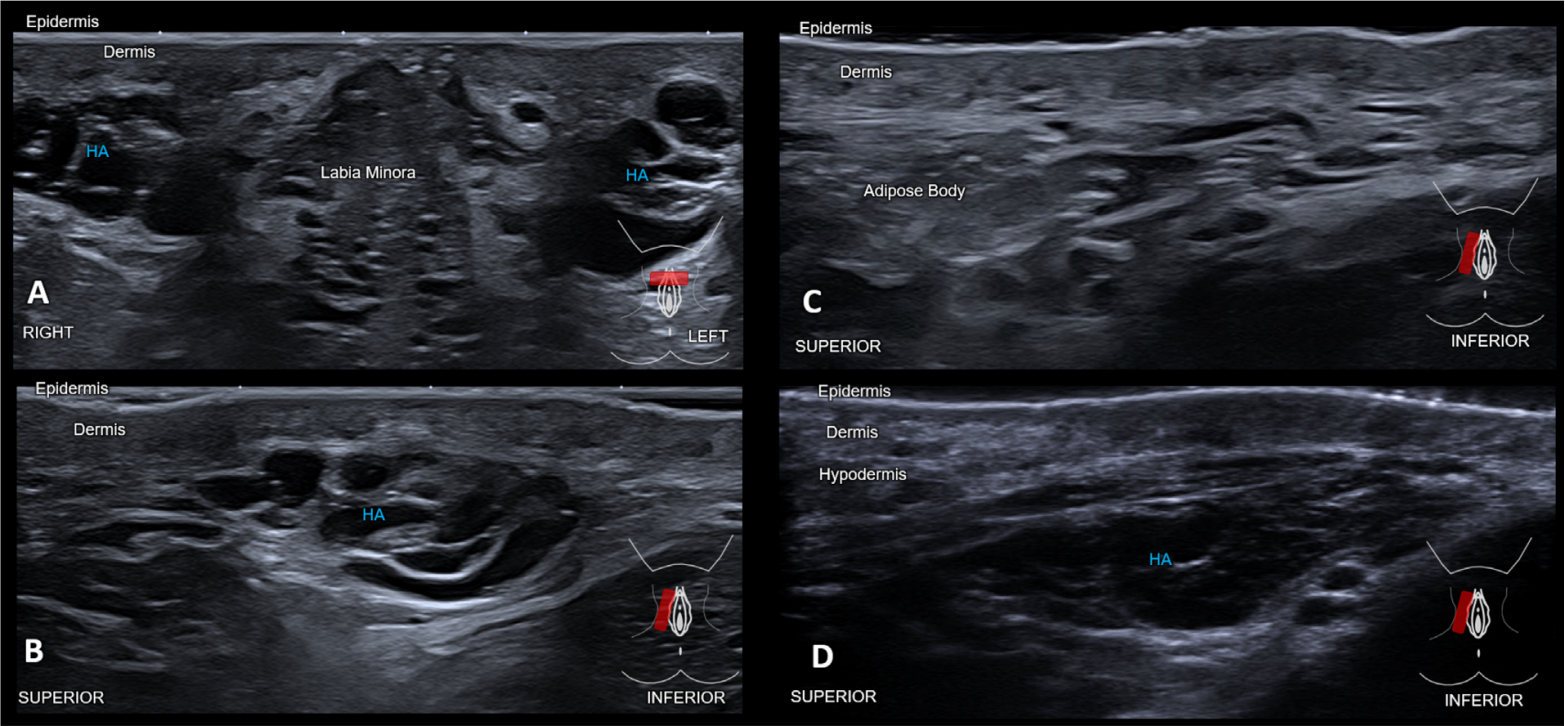
High‐frequency ultrasound (HFUS) images of hyaluronic acid (HA) filler in the labia majora. B‐mode 24 MHz images obtained immediately after injection (**A, B**) show well‐defined anechoic lobulated HA deposits located beneath the superficial fascia. Images obtained approximately 80 days after treatment (**C, D**) demonstrate similar distribution and homogeneous echogenicity, with preserved delineation of the fascial and subcutaneous planes.

High‐frequency ultrasound (HFUS) allowed clear visualization of HA filler deposits in the labia majora. Immediately after injection, HA appeared as well‐defined anechoic lobulated structures beneath the superficial fascia, with smooth contours and thin internal septa (Figure 3A,B). No internal vascular flow was detected on color Doppler, confirming its avascular nature and correct positioning within the subcutaneous plane. At approximately 80 days after treatment, HFUS demonstrated similar distribution and homogeneous echogenicity, with preserved delineation of fascial and subcutaneous structures (Figure 3C,D).

#### Calcium Hydroxyapatite

Calcium hydroxyapatite (CaHA) is used for treating tissue laxity and volume loss in the labia majora, promoting neocollagenesis and dermal remodeling over time.

**Figure 4:**
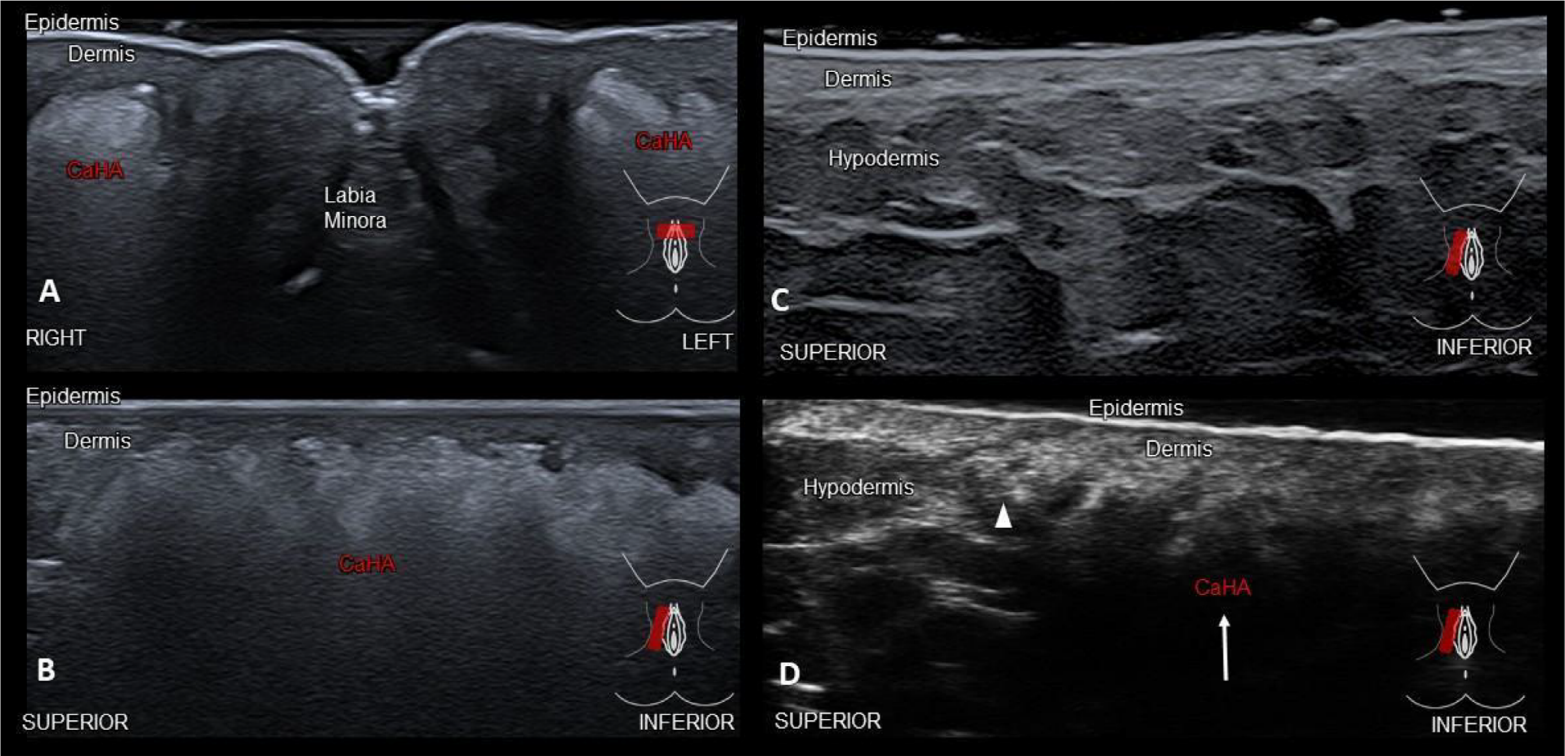
B‐mode 24 MHz images of calcium hydroxyapatite (CaHA) injection in the labia majora. (**A, B**) Immediately after injection showing hyperechoic deposits with coarse‐grain texture and posterior acoustic shadowing. (**C, D**) Images obtained approximately 80 days after treatment showing thickened dermis, blurred dermis–hypodermis interface (arrowhead), and persistent CaHA deposits with posterior acoustic shadowing (arrow).

On HFUS, immediately after injection, CaHA appeared as hyperechoic deposits with variable posterior acoustic shadowing. Depending on dilution and distribution, the ultrasonographic appearance may range from a coarse‐grain “snowfall” pattern to a more diffuse “cloudy” pattern (Figure 4A,B).

At approximately 80 days, persistent punctate hyperechoic foci were observed, along with dermal thickening and CaHA at the dermis–hypodermis interface, contributing to the altered echostructure (Figure 4C,D).

#### Polydioxanone Threads

**Figure 5:**
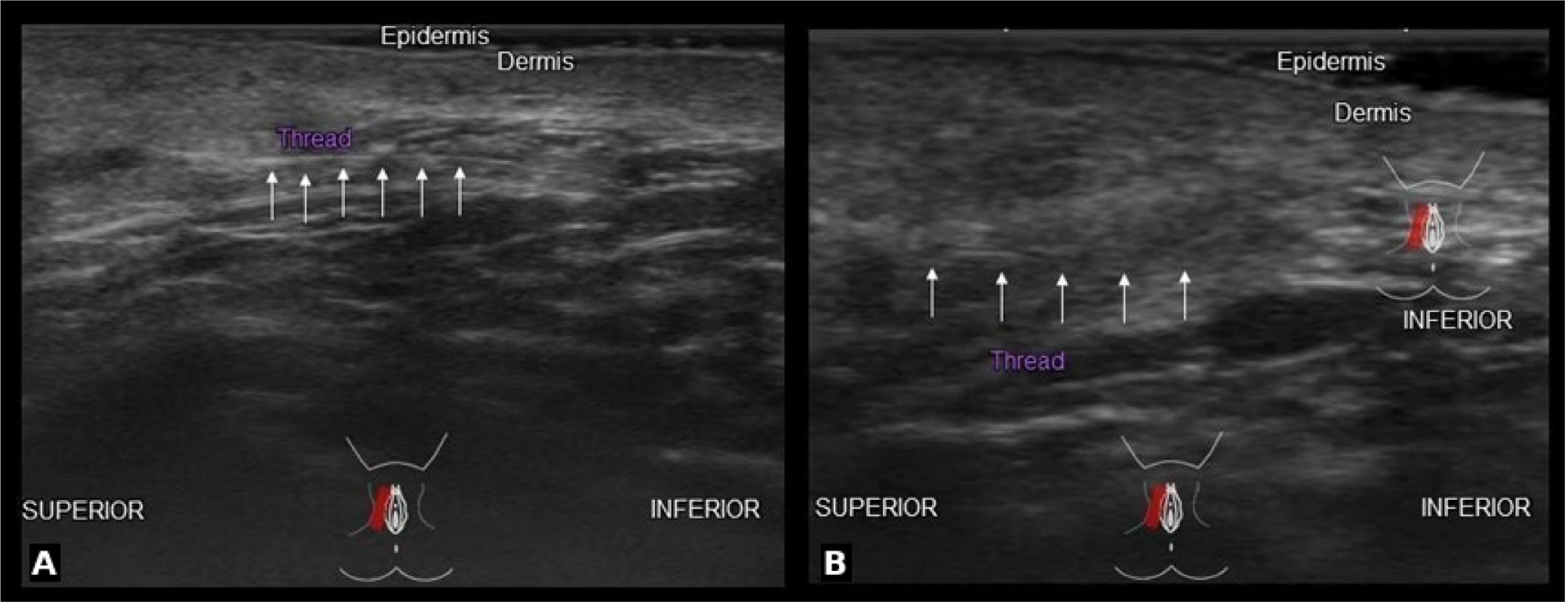
B‐mode 24 MHz images of polydioxanone (PDO) thread in the labia majora. (A, B) Immediately after insertion showing hyperechoic cylindrical structures with posterior acoustic shadowing in the subdermal layer (arrows).

Absorbable polydioxanone (PDO) threads are indicated for genital tissue tightening and collagen stimulation in patients presenting mild‐to‐moderate laxity of the labia majora. HFUS revealed linear hyperechoic bands on longitudinal views and round hyperechoic foci on transverse sections, where posterior acoustic artifact is more typically appreciated, corresponding to the inserted threads in the subdermal layer (Figure 5A,B).

#### Polymethyl Methacrylate (PMMA)

Polymethyl methacrylate (PMMA) is a permanent filler used in some cases for long‐lasting volumetric correction of the labia majora, though it is less commonly employed due to potential complications.

**Figure 6:**
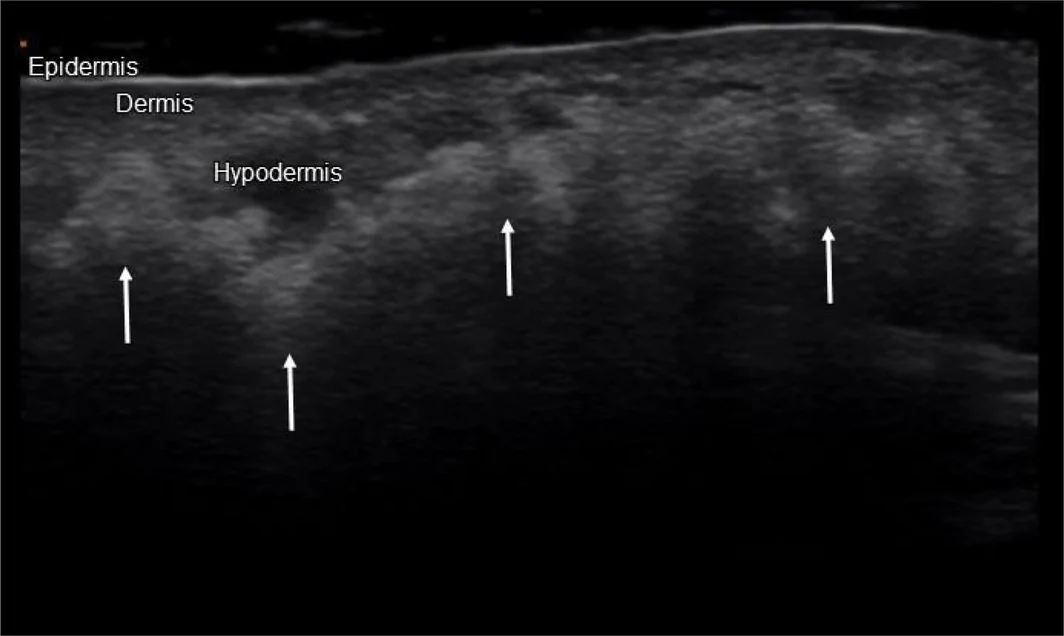
B‐mode 18 MHz image of polymethyl methacrylate (PMMA) in the labia majora showing hyperechoic deposits with hyperechoic spots and mini comet‐tail artifact (arrowheads) in the subcutaneous tissue.

On HFUS, PMMA appeared as multiple hyperechoic deposits/dots within the subcutaneous tissue, with mini comet‐tail artifact, consistent with its characteristic sonographic pattern (Figure 6).

#### Mesotherapy: Injectable Lipolytic Substances

Injectable lipolytic substances such as deoxycholic acid and phosphatidylcholine are used to reduce localized adiposity of the mons pubis or suprapubic region.

**Figure 7:**
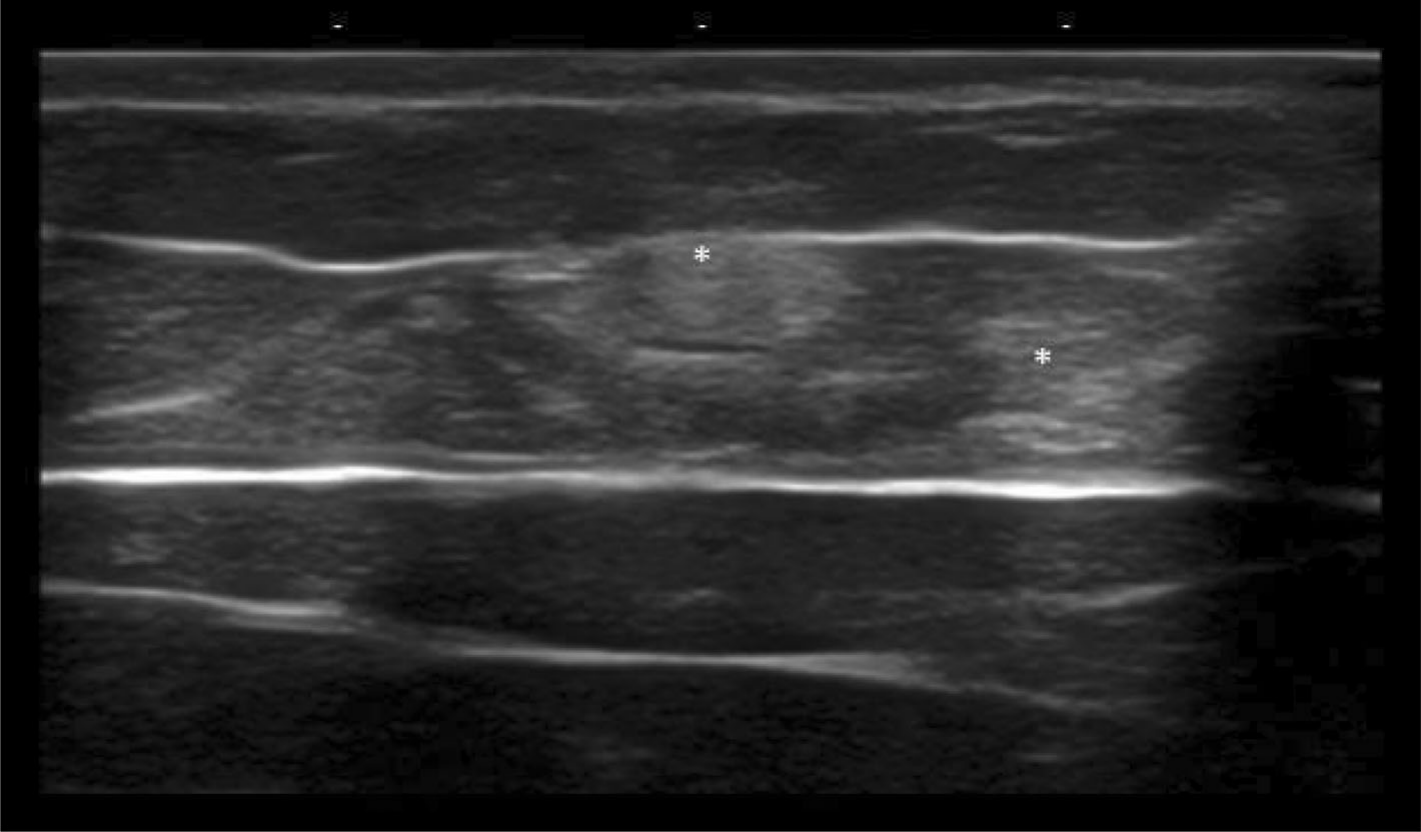
B‐mode 18 MHz image showing focal hyperechoic areas within the subcutaneous fat of the pubic region, consistent with panniculitis related to lipolytic injections (*).

Post‐treatment HFUS scans showed focal hyperechoic areas within the subcutaneous fat layer, compatible with inflammatory panniculitis at the injection sites (Figure 7).

### Complications and Other Pathological Findings

High‐frequency ultrasound (HFUS) also proved valuable for the identification and characterization of complications following genital rejuvenation procedures. The most frequent findings included superficial nodules, dermal thickening, and localized subcutaneous irregularities. In patients treated with hyaluronic acid fillers, well‐defined hypoechoic or anechoic rounded structures were observed just below the dermis, corresponding to areas of superficial infiltration or focal accumulation of material.

Inflammatory processes such as panniculitis and hidradenitis suppurativa were identified based on distinct ultrasonographic features. Panniculitis was characterized by alterations in the subcutaneous fat, including hyperechogenicity and blurring of adipose lobules or septal thickening.^11^ In contrast, hidradenitis suppurativa was identified by dermal thickening, pseudocysts, fluid collections, and, in some cases, fistulous tracts/tunnels connected to widened hair follicles.^12^ Folliculitis was characterized by hair follicle dilation and surrounding hypoechoic perifollicular inflammatory changes.

In addition to these benign complications, HFUS also facilitated the differentiation between inflammatory or infectious conditions and other nodular lesions, such as metastatic or neoplastic involvement, reinforcing its diagnostic value in postprocedural assessment.

## Discussion

This study demonstrates the applicability of HFUS in the anatomical evaluation and monitoring of female genital rejuvenation procedures, a rapidly expanding area that is still underexplored in terms of image documentation. While transperineal vulvar ultrasound has been increasingly standardized for the assessment of benign, inflammatory, and malignant vulvar disease,^13^ high‐frequency ultrasound is now recognized as a first‐line tool to personalize minimally invasive aesthetic procedures and manage filler‐related complications;^14^ systematic ultrasound descriptions focused on genital aesthetic rejuvenation with fillers, biostimulators, and absorbable threads are still lacking. Thus, our findings contribute to filling an important gap in dermatological, gynecological, and aesthetic practice.

High‐frequency ultrasound has evolved from a niche imaging resource into an integral component of minimally invasive aesthetic practice. In facial aesthetics, dermatologic ultrasound enables real‐time visualization of skin layers and the SMAS, preprocedural vascular mapping of high‐risk territories, and material‐specific identification of previously injected fillers, thereby improving planning and complication management.^15^, ^16^ Prospective data also suggest that preprocedural vascular mapping with ultrasound can translate into tangible clinical benefits, such as a significant reduction in bruising after dermal filler injections.^17^ Within this broader movement toward image‐guided aesthetic interventions, extending high‐frequency ultrasound (HFUS) to female genital rejuvenation represents a logical and timely next step.

Building on existing applications of transperineal vulvar ultrasound for vulvar disease assessment^13^ and on the established role of HFUS in aesthetic practice for procedural guidance, material identification, and complication documentation in other anatomical regions,^14^, ^18^ our series consolidates these principles into a pragmatic, genital‐focused imaging framework. Specifically, we propose a structured approach for baseline anatomical mapping and postprocedural monitoring, integrating key landmarks and representative sonographic appearances after fillers, biostimulators, and absorbable threads. This framework is intended to support consistent reporting and interdisciplinary communication across dermatologic, gynecologic, radiologic, and aesthetic settings.

Beyond anatomical mapping, HFUS also enables material‐specific pattern recognition. Distinct echogenic signatures have been consistently described for major injectable materials, allowing their organization into practical sonographic categories that support identification and differentiation of fillers and other implants.^19^ Recent reviews synthesize this body of evidence and position HFUS as a high‐resolution, first‐line imaging modality in aesthetic practice, capable of distinguishing filler and non‐filler materials, mapping previous procedures, diagnosing complications, and guiding real‐time interventions such as hyaluronidase injection or abscess drainage.^20^

Accordingly, this study provides a descriptive overview of how HFUS can be integrated into female genital rejuvenation by illustrating its use in pre‐procedural assessment, intra‐procedural guidance, post‐procedural follow‐up, and the recognition of treatment‐related changes, age‐related tissue alterations, and potential complications. Across a small series of real‐world cases, we outline a practical scanning approach for the vulvar region, highlight key anatomical landmarks, and document representative sonographic appearances after procedures with HA fillers, CaHA‐based biostimulators, and absorbable PDO threads. A strength of this work is its focus on an anatomically and culturally sensitive area that remains underrepresented in imaging literature, offering an initial visual and procedural framework that may assist dermatologists, gynecologists, radiologists, and aesthetic practitioners who wish to incorporate HFUS into their practice.

The subepidermal low‐echogenic band (SLEB) is commonly associated with photoaging; however, it has also been described in non–photoexposed areas and younger individuals, suggesting a multifactorial etiology related to intrinsic aging, anatomical site, and dermal water content.^21^, ^22^ Therefore, its presence in the genital region should be interpreted with caution, particularly in the absence of a control group. However, the descriptive, single‐center design, limited sample size, single‐operator acquisition and interpretation of the ultrasound examinations, variability in clinical presentations, and absence of standardized clinical outcome measures mean that these observations should be interpreted as exploratory and hypothesis‐generating rather than definitive. Taken together, these preliminary observations may serve as a starting point for future prospective studies aimed at defining the role of HFUS in female genital rejuvenation practice.

## Conclusion

HFUS appears to be a useful adjunct in female genital rejuvenation, offering high‐resolution assessment of anatomy, injected materials, and treatment‐related changes. Based on this small descriptive series, HFUS may help clinicians plan, monitor, and troubleshoot minimally invasive procedures in an area of the imaging literature that remains underdocumented.

## Data Availability

The data that support the findings of this study are available from the corresponding author upon reasonable request.
